# Assessment of the plasticity of dry granulated particles (mini-tablets) and its relationship to their tabletability

**DOI:** 10.1016/j.ijpx.2025.100351

**Published:** 2025-07-02

**Authors:** Maryam Tofiq, Göran Alderborn, Josefina Nordström, Ann-Sofie Persson

**Affiliations:** Department of Pharmaceutical Biosciences and the Swedish Drug Delivery Center (SweDeliver), Uppsala University, Box 591, SE-751 24 Uppsala, Sweden

**Keywords:** Mini-tablets, Dry granulation, Indentation hardness, Fracture force, Powder compression, Compression parameters, Tablet tensile strength

## Abstract

Mini-tablets of different proportions of *α*-lactose monohydrate (LAC) and microcrystalline cellulose (MCC) were prepared by uniaxial compaction and served as surrogate granules. The inverted Adams coefficient i.e., *α*^−1^ was derived from bulk mini-tablet compression data and used as an indication of granule plastic deformation. The correlation of the parameter to single granule deformability assessed from uniaxial single mini-tablet compression and macro-indentation hardness was investigated. Furthermore, the relationship between the plastic deformation parameters and the tabletability of mini-tablets were evaluated. An increased MCC concentration resulted in an increased indentation hardness and deformability of the mini-tablets, but no correlation was found between indentation hardness and the *α*^−1^ coefficient. Thus, the plastic deformation expressed during powder compression showed no relationship to the single specimen indentation hardness and plastic deformability. An increased indentation hardness tended to correspond to an increased tablet tensile strength, while the opposite applied for the *α*^−1^ coefficient. The trend of increased tablet tensile strength with higher MCC concentration was broken at the highest MCC concentration, i.e., for mini-tablets showing very limited fragmentation. It was concluded that granule plastic deformation is a key property for granule tabletability. It is suggested that granule plastic deformation should be assessed during granule engineering.

## Highlights

• Plastic deformation of dry granulated particles is a key property for their tabletability.
• Limited granule fragmentation may contribute to an improved tabletability.
• The plasticity of the granule forming particles is critical for the granule plasticity.
• Single granule plasticity did not correlate to plastic deformation during powder compression.
• Analytical powder compression is the preferred approach to measure granule plastic deformation.

## Graphical abstract

**Figure:**
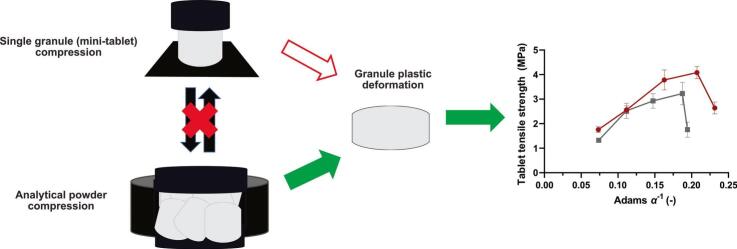

## List of symbols

*Symbol*: *Meaning (*
*Unit)*
*A*: Heckel intercept
  (–)
*C*: Engineering strain
  (–)
*D*: Ball indenter diameter (
  mm)
*D*_t_: Tablet diameter (
  mm)
*d*: Diameter of the circular indent (
  mm)
*E*_H_: In die porosity of tablets, Heckel equation (
  %)
*E*_g_: Intra-granular porosity (
  %)
*E*_I_: Indent tablet porosity (
  %)
*E*_t_: Geometrical tablet porosity (
  %)
*ƒ*_MCC_: Concentration of MCC in granulated powders
  (–)
*F*_I_: Indentation force (
  N)
*F*_g axial_: Granule fracture force on axial direction (
  N)
*F*_t_: Tablet fracture force (
  N)
*HB*: Indentation hardness
  (–)
*h*_0_: Initial powder bed height (
  cm)
*h*: Current powder bed height (
  cm)
*h*_t_: Tablet height (
  mm)
*K*: Single granule plastic deformation (
  N/mm)
*k*: Heckel slope (
  MPa^−1^)
*P*: Applied pressure (
  MPa)
*P*_g_: Granule production pressure (
  MPa)
*P*_y_: Heckel yield pressure (
  MPa)
*α*: Adams friction coefficient
  (–)
*ε*_n_: Natural strain, Adams equation
  (–)
*ρ*_app_: Apparent particle density (
  g/cm^3^)
*ρ*_g bulk_: Granule bed bulk density (
  g/cm^3^)
*ρ*_t_: Tablet density (
  g/cm^3^)
*τ*_0_: Adams single granule strength (
  MPa)
*σ*_t_: Tablet tensile strength (
  MPa)

## 1 Introduction

The compression and compaction properties of powders prepared by dry granulation technologies have been the focus of several investigations reported in the literature, a review of a significant part of this literature can be found elsewhere (Sun & Kleinebudde, 2016). Since a tablet must possess a sufficient tensile strength to ensure tablet integrity during handling, a key property of a dry granulated powder is its tabletability, i.e. the ability to form tablets during powder compression as defined in USP <1062> (2017). In addition to the tabletability, the tableting performance of dry granulated powders is commonly also studied by the ratio between the tensile strengths of tablets formed from the dry granulated particles and those formed from the ungranulated particles (Herting & Kleinebudde, 2008; Nordström & Alderborn, 2015), i.e. the loss of tabletability (LoT). A compression mechanism of importance for the LoT is granule fragmentation (Tofiq et al., 2022a; Tofiq et al., 2025). The propensity of granules to fragment is dependent on their fracture strength (Tofiq et al., 2025). Not only for the compression properties of granules but also during their technical handling more generally, such as during transport and mixing, the fracture or failure strength of granules is important. There is thus a need to have methods that can assess granule fracture strength. Granules can vary considerably in shape, from spherical particles to irregular objects but pharmaceutical granules prepared by wet or dry granulation are mostly irregular. For such granules a large variability in derived strength data may be obtained (Adams et al., 1994) affecting the possibility to discriminate properties between different granules. An alternative approach is instead to use powder compression as a means to derive indications of particle failure strength, i.e. to calculate single particle properties from macroscopic powder compression properties. Single specimen testing is typically done on relatively few objects. In contrast, a compression parameter derived from powder compression data is based on the analysis of a large number of particles of varying sizes and shapes through a simple experiment.

Adams et al. (Adams et al., 1994) derived an equation to calculate a single granule failure strength from powder compression data. A micro-mechanical mechanistic model focusing on the failure of granules located in load bearing columns during volume reduction of the powder was used and a compression equation was developed by which failure strength of the single granules, commonly denoted *τ*_0_, can be calculated. It was also shown that the*τ*_0_ parameter correlated with a parameter obtained by the Kawakita equation (Adams & McKeown, 1996; Adams et al., 1994; Yap et al., 2008), also reported later by Nicklasson and Alderborn (Nicklasson & Alderborn, 2000) and Nordström et al. (Nordström et al., 2008). The Adams equation also includes a second compression parameter, a pressure or friction coefficient here denoted *α*.

In a recent study (Tofiq et al., 2022b), the compression properties of a series of dry granulated powders were investigated. The basic description used of the compression process was the relationship between the engineering strain of the powder and the uniaxially applied compression pressure, giving a compression curve which initially increased nearly linearly and thereafter bended markedly - referred to as the macroscopic stiffening phase - and finally slowly approached a maximum degree of compression. Depending on the composition of the granules their compression properties could be differentiated by different sharpnesses of the bending of the compression curve, i.e. different stiffening rates. It was further found that the Adams equation linearized the compression process in the compression pressure range (about 10–50 MPa) during which the compression curves bended. The slope of this linear part is the Adams *α* coefficient which thus gives an indication of the rate of macroscopic stiffening of the powder. It was argued that the stiffening rate was depended on the plasticity of the granules, a conclusion based on mechanistic understanding of the compression process, i.e. the compression occurs in a sequence of different phases controlled by different compression mechanisms similar to those proposed earlier for wet granulated particles (Johansson & Alderborn, 2001; van der Zwan & Siskens, 1982). Initially, rearrangement of granules occur until a jamming point is reached and thereafter the compression is dominated by granule fragmentation. As indicated experimentally (Skelbæk-Pedersen et al., 2021; Tofiq et al., 2025) granule fragmentation ceases to occur already at a low applied pressure, about 10 MPa. Thereafter, during the macroscopic stiffening, granule plastic deformation must be the mechanism controlling the compression process. Finally, at high applied pressures a low rate of compression will take place and proposed to be due to ceased granule plastic deformation and a transition to an elastic deformation of a stiff compact. Since the slope of the linear part of the Adams profile reflects the granule plastic deformation phase, the Adams friction coefficient (*α*) could serve as an indication of the granule plasticity or plastic stiffness. This interpretation of the *α* coefficient was supported by a strong positive correlation between the inverted coefficient and the tensile strength of tablets formed form the granulated powders (Tofiq et al., 2022a).

The Adams *α* coefficient deserves to be further explored as a potentially valuable variable characterizing a significant compression mechanism of granules. Of specific interest is to study if the coefficient correlates to the plastic deformability of the granules as assessed by single specimen analysis and we now ask the question if single granule plasticity data correlates with the coefficient and can be used to predict plastic deformation of granules during powder compression. Of interest is also to study if any of these plasticity parameters are linked to the tabletability of the granules. To simplify the measurements of the plasticity of single granules, two types of surrogate granules are used, i.e., mini-tablets and flat-faced tablets formed by powder compaction but without any subsequent milling. In addition, compression parameters were also derived for the mini-tablets by compression of an assembly of mini-tablets in a tableting die.

## 2 Materials and methods

### 2.1 Materials

Microcrystalline cellulose (Avicel PH 101, FMC Biopolymer, U.S.A., abbreviated MCC) and crystalline *α*-lactose monohydrate (Pharmatose 200 mesh, DMV, Veghel, The Netherlands, abbreviated LAC) powders, and binary mixtures of MCC and LAC in the proportions 75:25, 50:50, and 25:75, were used as granule forming fine powders. Magnesium stearate (Sigma-Aldrich, Sweden, abbreviated MgSt) was used as a lubricant. The preparation of the binary mixtures and the characterization of the apparent particle density, unsettled particle density, and volume specific surface area of the fine powders are described in a previous paper (Tofiq et al., 2022b).

### 2.2 Preparation of mini-tablets and indentation test tablets

Two types of tablets with well-defined shapes were used as surrogates for typical irregular granulated particles, namely mini-tablets and flat faced tablets, the latter used for indentation hardness testing and henceforth referred to as indentation test tablets.

Mini-tablets were manually compacted from the fine powders at 50 and 100 ± 5 MPa using a single punch tablet press (Korsch EK0, Germany) as described earlier (Tofiq et al., 2025). The punches used during compaction had a concave shape and a diameter of 3.0 mm. The amount of powder compacted was adjusted to give mini-tablets with a height of 3.0 ± 0.2 mm and a diameter of 3.0 ± 0.06 mm. The weight was of 21 ± 1 mg (for mini-tablets compacted at 50 MPa) and 24 ± 1 mg (for mini-tablets compacted at 100 MPa). Prior to their subsequent characterization, the mini-tablets were stored for at least seven days at room temperature (20 °C) in a humidity controlled room with a relative humidity of ∼33%. The unsettled bulk density (*ρ*_g bulk_) and the intra-granular porosity (*E*_g_) were measured, as described in (Tofiq et al., 2025).

Circular flat-faced indentation test tablets with a diameter of 11.3 mm were compacted of all ungranulated fine powders and subsequently subjected to macro-indentation testing. The tablets were compacted by uniaxial compaction of 0.4 g fine powder material in a MgSt pre-lubricated die using a materials testing instrument (Zwick Z100, Zwick/Roell GmbH & Co, Ulm, Germany) equipped with a 10 kN load cell. A linear loading rate of 10 mm/min was used, and for each composition, the applied compaction pressure was adjusted to achieve tablet porosities (*E*_I_) corresponding to the intra-granular porosities of the mini-tablets produced at 50 and 100 MPa. Four tablets of each powder material and for each porosity were prepared.

### 2.3 Fracture resistance and plastic deformation of mini-tablets

A materials testing instrument (Zwick/Roell Z0.5, Germany), equipped with a 500 N load cell, was used to measure the plastic deformation and the fracture force of a single mini-tablet while loaded in the axial direction, i.e. in the same direction as the movement of the upper punch during compaction. The mini-tablets (n = 20) were compressed uniaxially at 0.1 mm/s using a 11.3 mm diameter flat-faced cylindrical movable upper probe. Before compression, the height and diameter of the mini-tablets were recorded (Litematic VL-50A, Mitutoyo, Japan). The axial fracture force (*F*_g axial_) was determined as the maximum force observed in the force-displacement profile, i.e., at the point where a distinct drop in the profile occurred, and the slope of the linear region (R^2^ > 0.986) of each profile was used as a measure of the plastic deformation (*K*). The displacement range used for determining the slope was selected visually from the force-displacement profile to be as broad as possible and varied depending on the granule composition. Each slope was calculated from a minimum of 500 data points.

### 2.4 Indentation hardness

The indentation tests were performed in a materials testing instrument (Zwick/Roell Z0.5, Germany) equipped with a 500 N load cell and a ball shaped indenter with a diameter (*D*) of 5 mm. Three separate dents were made on each tablet surface, resulting in a total of 12 dents per material composition and tablet porosity. The force (*F*_I_) applied on each tablet was approximately two-thirds of the tablet fracture force (*F*_t_) (Sun et al., 2018), which was measured diametrically with a hardness tester (PharmaTest PTB511E, Hainburg, Germany) at a linear loading rate increment of 20 N/s. During indentation, the *F*_I_ was applied at a speed of 0.1 mm/s, and the *F*_I_ was held for 180 s before unloading. After unloading, the tablet surface was gently brushed with a soft colored powder to create a contrast, facilitating the measurement of the dent diameter. An image of the tablet surface was captured using a light microscope (Zeiss AxioCam ICc 5, Carl Zeiss AB, Sweden), and the diameter of the circular dent (*d*) was measured using the length measurement tool available in the microscope software (AxioVision, Carl Zeiss AB, Sweden). From the dent and indenter diameters, the Brinell indentation hardness (*HB)* was calculated using the following equation (Hutchings, 2011):

(1) $$\mathrm{HB}=\frac{2F_{I}}{\left(\mathrm{\pi D}\right)\left(D-\sqrt{D^{2}-d^{2}}\right)}$$

### 2.5 Powder compression analysis of mini-tablets

Approximately 0.4 g of mini-tablets (corresponding to up to 20 mini-tablets) were compressed using uniaxial compression with a materials testing instrument (Zwick Z100, Zwick/Roell GmbH & Co, Ulm, Germany) equipped with flat-faced circular (11.3 mm diameter) punches. A linear (constant) loading rate of 10 mm/min up to 300 MPa was applied. Prior to compression, the punches and the die surfaces were lubricated with 1% *w*/*v* MgSt suspension in ethanol and dried. Five independent compressions were performed for each composition. The series of obtained force-displacement curves were first corrected for elastic system deformation as described earlier (Nordström et al., 2008) and thereafter the force-displacement of the loading stage was fitted to the Heckel and Adams equations, as detailed below.

First, the in-die force-displacement curves were transformed into porosity-pressure relationships and plotted according to the Heckel equation (Heckel, 1961):

(2) $$\ln\left(\frac{1}{E_{H}}\;\right)=\mathrm{kP}+A$$

where *E*_H_ is the in-die porosity, *P* is the applied pressure, and *k* and *A* are constants. By plotting $\ln\left(\frac{1}{E_{H}}\;\right)$ as a function of *P*, linear relationships were obtained in the pressure range 65 to 280 MPa. The constant *k* was determined using linear regression (R^2^ > 0.999). The apparent yield pressure (*P*_y_) was calculated from the reciprocal of *k* and used as a measure of the primary particle plasticity as described earlier (Tofiq et al., 2022b).

Secondly, the force-displacement curves were transformed into natural strain-pressure relationships and plotted according to the Adams equation (Adams & McKeown, 1996):

(3) $$\ln P=\ln\;\frac{\tau_{0}}{\alpha }+\alpha \varepsilon_{n}+\ln\left(1-e^{\left(-\alpha \varepsilon_{n}\right)}\right)$$

where *P* is the applied pressure, *τ*_0_ is a parameter representing the single mini-tablet failure strength, *α* is a friction or pressure coefficient. *ε*_n_ is the natural strain that is calculated as:

(4) $$\varepsilon_{n}=\ln\left(\frac{h_{0}}{h}\right)$$

where *h*_0_ is the initial mini-tablet bed height in the die, and *h* is the mini-tablet bed height at the applied pressure *P*. By plotting ln *P* as a function of *ε*_n_, linear relationships were obtained in the pressure range 20–50 MPa (R^2^ > 0.999), which was used for calculating the *τ*_0_ and *α* parameters.

Thirdly, the force-displacement curves were transformed in to engineering strain (*C*) – pressure (*P*) relationships. *C* was calculated as the ratio between (*h*_0_−*h*) and *h*_0_, where *h*_0_ is the initial mini-tablet bed height calculated from the unsettled granule bulk density, and *h* is the mini-tablet bed height at the applied pressure *P*.

### 2.6 Compaction of mini-tablets and tablet characterization

Approximately 0.4 g of mini-tablets were compacted uniaxially at 100, 200, and 300 MPa using the materials tester at the same settings as described in Section *2.5*. For each composition and each compaction pressure, four individual tablets were produced.

Immediately after ejection, the tablet weight and tablet height (*h*_t_) and diameter (*D*_t_) were determined (Litematic VL 50A, Mitutoyo Corp., Kanagawa, Japan). The geometrical tablet porosity (*E*_t_) was thereafter calculated using the following equation:

(5) $$E_{t}=1-\frac{\rho_{t}}{\rho_{\mathrm{app}}}$$

where $\rho_{t}$ is the tablet density calculated from the tablet weight and tablet volume calculated from the tablet dimensions i.e. height and diameter, and $\rho_{\mathrm{app}}$ is the apparent density of the ungranulated fine powder.

The force required to break the tablets (*F*_t_) was determined by compressing single tablets diametrically until breakage using a PharmaTest instrument (PTB511E hardness tester, Hainburg, Germany) at a linear loading rate increment of 20 N/s. The tablet tensile strength (*σ*_t_) was calculated as (Fell & Newton, 1970):

(6) $$\sigma_{t}=\frac{2F_{t}}{\pi h_{t}D_{t}}.$$

### 2.7 Statistical analysis

Statistical analyses were conducted using either a one-way analysis of variance (ANOVA) test followed by Tukey post hoc test or paired *t-test*. The significance level was set to 95%.

## 3 Results

### 3.1 Densities

**Fig. 1:**
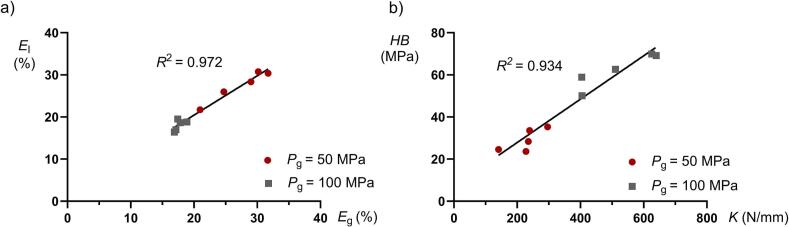
The a) indentation test tablet porosity as a function of the intra-granular porosity and b) indentation hardness as a function of the axial single mini-tablet plastic deformation for all compositions, compacted at 50 (red dots) and 100 MPa (gray squares). (For interpretation of the references to colour in this figure legend, the reader is referred to the web version of this article.)

The bulk density (*ρ*_g bulk_, Table 1) for the mini-tablets produced at 50 MPa was slightly lower compared to the mini-tablets produced at 100 MPa. In addition, the porosity (Fig. 1a) was generally higher for the mini-tablets produced at 50 MPa than for those produced at 100 MPa. Thus, the difference in bulk density can be attributed to the varying porosity of the mini-tablets. For the mini-tablets compacted at 50 MPa, there was a tendency for the porosity to decrease with decreasing *ƒ*_MCC_, while for mini-tablets compacted at 100 MPa, no change in porosity with *ƒ*_MCC_ was obtained.

**Table 1:** Characteristics of all mini-tablets compacted at 50 and 100 MPa. Relative standard deviations in percent (%RSD) provided within parentheses.

Material	*ρ*_g bulk_^a^ (g/cm^3^)	*HB*^b^ (MPa)	*F*_g axial_^c^ (N)	*K*^d^ (N/mm)	*P*_y_^e^ (MPa)	*τ*_0_^f^ (MPa)	*α*^f^ (−)
100% *f*_MCC_ 50 MPa	0.503 (0.26)	35.32 (2.24)	49.9 (11.5)	296.3 (10.3)	87.1 (1.41)	5.741 (7.15)	4.32 (2.38)
100 MPa	0.593 (1.66)⁎	69.92 (5.10)	93.9 (8.33)⁎	625.6 (9.93)	90.8 (1.05)	7.532 (14.9)	5.14 (5.92)
75% *f*_MCC_ 50 MPa	0.478 (1.25)	33.60 (3.65)	27.8 (13.8)	239.2 (12.5)	110.3 (3.82)	3.383 (7.84)	4.82 (2.43)
100 MPa	0.607 (1.20)⁎	69.18 (1.72)	70.5 (11.6)⁎	639.9 (16.2)	116.3 (1.76)	7.977 (11.5)	5.34 (3.70)
50% *f*_MCC_ 50 MPa	0.536 (1.88)	28.34 (3.77)	18.9 (18.9)	235.4 (17.3)	127.2 (0.62)	2.224 (2.56)	6.14 (0.55)
100 MPa	0.604 (2.29)⁎	62.63 (4.65)	39.0 (15.7)⁎	510.7 (17.9)	131.9 (0.98)	3.016 (12.5)	6.78 (3.30)
25% *f*_MCC_ 50 MPa	0.491 (1.74)	24.57 (7.29)	5.88 (33.4)	140.9 (31.8)	154.8 (0.76)	0.118 (6.84)	8.94 (0.85)
100 MPa	0.589 (2.68)⁎	58.90 (4.76)	18.1 (33.0)⁎	404.1 (29.8)	159.3 (1.58)	0.782 (10.5)	8.97 (1.81)
0% *f*_MCC_ 50 MPa	0.504 (1.58)	23.62 (11.7)	7.22 (28.3)	227.7 (25.5)	183.2 (0.90)	0.016 (27.1)	13.66 (2.37)
100 MPa	0.609 (1.75)⁎	50.10 (5.44)	14.5 (42.5)⁎	405.2 (31.2)	199.2 (1.50)	0.065 (30.5)	13.54 (2.90)

⁎ The data are presented in Tofiq et al. (Tofiq et al., 2025).

a Mini-tablet bed bulk density (*ρ*_g bulk_; *n* = 3).

b Indentation hardness (*HB*; *n* = 12).

c Mini-tablet fracture force in axial direction (*F*_g axial_; *n* = 20).

d Single mini-tablet plastic deformation parameter (*K*; *n* = 20).

e Heckel compression parameter (*P*_y_; *n* = 5).

f Adams compression coefficients (*τ*_0_ and *α*; *n* = 5).

The porosity of the series of flat-faced tablets prepared specifically for the indentation experiments (the indentation test tablets) correlated well with the porosity of the mini-tablets (Fig. 1a). Thus, the indentation test tablets had a similar variation in micro-structure as the mini-tablets. This range in porosity, about 15–30%, is similar to the porosity range of dry granulated particles of the same compositions studied earlier (Tofiq et al., 2022b) prepared by slugging-milling-sieving.

### 3.2 Fracture resistance and deformability of mini-tablets

Single mini-tablets were subjected to uniaxial compression as a means to derive indications of their fracture resistance and deformability. For the mini-tablets prepared at 100 MPa the force-displacement profiles are presented elsewhere (Tofiq et al., 2025), indicating that at low applied forces, the profiles were non-linear but became approximately linear at higher forces, i.e., the mini-tablets showed an elasto-plastic behavior. The force-displacement profiles for the mini-tablets produced at 50 MPa showed a similar appearance (data not shown). The axial fracture force (*F*_g axial_) of the mini-tablets increased with a rise in *ƒ*_MCC_ and with decreased porosity (Table 1). An increased *ƒ*_MCC_ and a decreased porosity tended to increase the slope of the force-displacement profiles (the plastic deformation parameter *K*), corresponding to a decreased deformability (increased plastic stiffness).

### 3.3 Indentation hardness

**Fig. 2:**
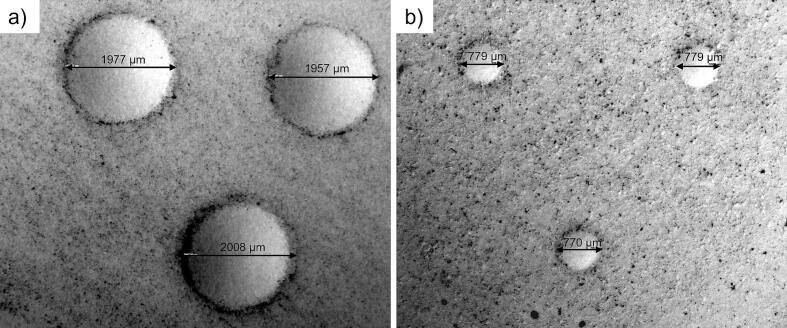
Examples of dents after indentation of indentation test tablets of a) 100% *f*_MCC_ and b) 0% *f*_MCC_ compacted at 100 MPa.

The indentation hardness (*HB*) (Table 1) were considerably higher for tablets prepared at 100 MPa compared to those at 50 MPa for all compositions, i.e., the porosity affected significantly the indentation hardness. Moreover, the *HB* showed a weak tendency to increase with increased *ƒ*_MCC_ for both high and low porosity indentation test tablets. Examples of dents from the indentation tests are given in Fig. 2.

A positive correlation (R^2^ = 0.934) was obtained between *HB* and the single mini-tablet plastic deformation parameter *K* (Fig. 1b), i.e. both parameters gave similar indications of the plasticity of the formed specimen.

### 3.4 Powder compression of mini-tablets

**Fig. 3:**
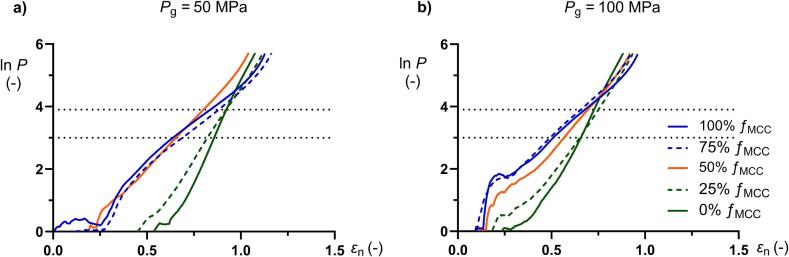
Typical Adams plots for all mini-tablet compositions compacted at 50 and 100 MPa. The dotted lines show the pressure region used in the linear regressions.

The Adams plots (Fig. 3) i.e., the natural strain as a function of compression pressure, showed a complex pattern at low compaction pressures, most obvious for the mini-tablets containing high *ƒ*_MCC_ and produced at the higher pressure. At higher compaction pressures the plots became generally nearly linear and the slope of the linear relationships varied depending on the granule composition. The Adams compression coefficients were thus calculated within a restricted range of compaction pressures, i.e., the linear region considered to correspond to the macroscopic stiffening phase of the compression profiles (Tofiq et al., 2022a; Tofiq et al., 2025). The Adams *τ*_0_ parameter generally increased with increased *ƒ*_MCC_ (Table 1), and the low porosity mini-tablets had somewhat higher *τ*_0_ than the more porous granules. For the compositions containing *ƒ*_MCC_ ≥ 50%, the *α* coefficient decreased with increasing *ƒ*_MCC_ (Table 1), and the more porous mini-tablets showed lower (*p* < 0.05) values than the less porous mini-tablets. For the compositions containing *ƒ*_MCC_ < 50%, the *α* coefficient remained independent of mini-tablet porosity.

The Heckel plots showed good linearity within the pressure ranges chosen for the calculation of the Heckel yield pressure *P*_y_ (plots not shown but were similar to earlier reported plots (Tofiq et al., 2022b) for irregular granules of the same compositions). The *P*_y_ (Table 1) decreased with increased *ƒ*_MCC_, and a lower *P*_y_ (*p* < 0.05) was obtained for the mini-tablets produced at 50 MPa compared to those produced at 100 MPa. The Heckel yield pressures were generally similar to the Heckel yield pressures obtained earlier (Tofiq et al., 2022b) for irregular granules of the same composition. Moreover, the *P*_y_ values were similar with, albeit somewhat higher, the corresponding values for the ungranulated powders (Tofiq et al., 2022b).

**Fig. 4:**
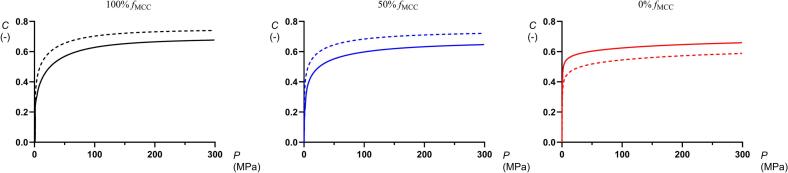
Typical engineering-strain (*C*) – pressure (*P*) profiles for two types of granules composed of 100% *f*_MCC_ (left), 50% *f*_MCC_ (middle) and 0% *f*_MCC_ (right). The solid lines denote the mini-tablets used in this study prepared at 50 MPa and the dashed lines denote dry granulated particles with a size fraction 500–710 μm reported earlier (Tofiq et al., 2022b).

The engineering strain – pressure profiles increased initially rapid at low applied pressures and started to bend after reaching the jamming transition. At about 100 MPa the profiles levelled out. Fig. 4 displays typical profiles for mini-tablets produced at 50 MPa of three of the compositions (100%, 50% and 0% *ƒ*_MCC_). For the engineering strain – pressure profiles for mini-tablets produced at 100 MPa, the reader is referred to Tofiq et al. (Tofiq et al., 2025). Some of the compression curves, i.e. for low porosity mini-tablets containing ≥50% *f*_MCC_, displayed a shoulder in the profiles probably due to a distinct jamming transition. A similar distinct shoulder was not obtained for the high porosity mini-tablets. For comparison, the compression profiles for dry granulated particles presented in (Tofiq et al., 2022b) were also plotted in Fig. 4. The character of the strain-pressure profiles of these dry granulated particles resembled the profiles of the mini-tablets. In the first part of compression process the profiles for the respective type of granular material overlapped but the strain at which the bending started differed. The curvature of the bending was similar between dry granulated particles and mini-tablets for each composition and thus, the final part of the profiles was shifted along the strain axis giving differences in the final degree of compression. For the 100% and 50% *f*_MCC_ the dry granulated particles gave a higher final degree of compression than the mini-tablets but for 0% *f*_MCC_ the converse applied. Thus, the compressibility was affected by the size and shape of the materials and their fragmentation during compression.

**Fig. 5:**
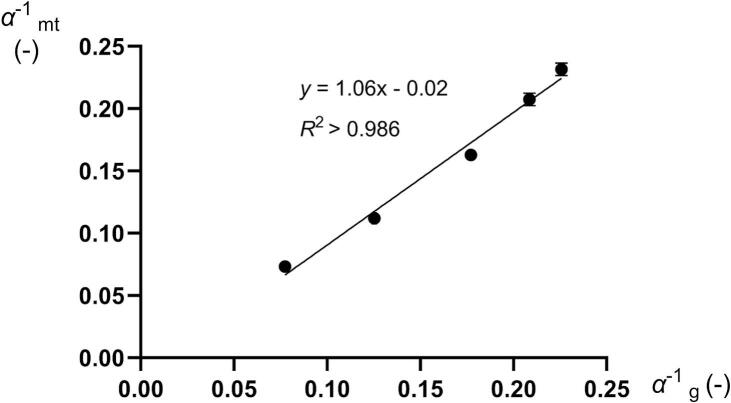
The inverted Adams coefficient for the mini-tablets) ($\alpha_{\mathrm{mt}}^{-1}$) as function of the inverted Adams coefficient for dry granulated particles prepared by slugging ($\alpha_{g}^{-1}$, (Tofiq et al., 2022b).

In Fig. 5, the inverted Adams coefficient *α*^−1^ of the mini-tablets reported in this study is plotted versus the inverted Adams coefficient *α*^−1^ for the dry granulated particles reported earlier (Tofiq et al., 2022b). For the mini-tablets, the coefficients were calculated in the pressure range 20–50 MPa while for the dry granulated particles, which gave linear Adams plots over a wider pressure range, the coefficients were calculated in the pressure range 10–50 MPa. A regression analysis gave a good correlation between these two coefficients with a slope of nearly one and an intercept of nearly zero. Thus, a nearly perfect relationship was obtained supporting the highly similar compression behavior in the macroscopic stiffening phase of the compression profiles.

In summary, the compression parameters for the studied mini-tablets depended on both their composition and porosity, in accordance with earlier findings for dry granulated particles (Tofiq et al., 2022b). The overall compression profiles differed between the two types of granular material suggesting that the size and shape of the single granules are of importance for their compression behavior. However, a great similarity was obtained in the region of the profiles characterized by a marked macroscopic stiffening.

### 3.5 Compactibility and tabletability

**Fig. 6:**
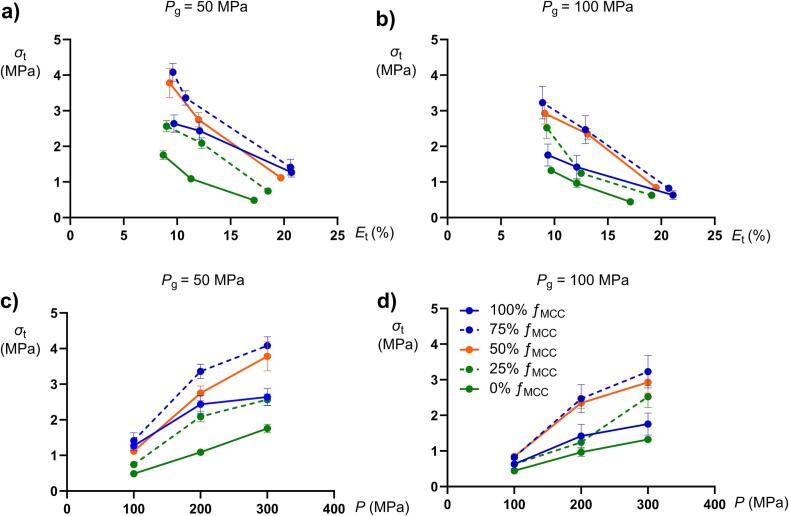
The tablet tensile strength (*σ*_t_) as a function of the a-b) tablet porosity (*E*_t_) and c-d) tableting pressure (*P*) for all mini-tablet compositions compacted at 50 and 100 MPa. The bars denote the standard deviations.

For each composition and tableting pressure, mini-tablets of high and low porosity gave practically the same tablet porosity (Fig. 6a-b). For tablets compacted at 100 MPa, an increased *f*_MCC_ increased the tablet porosity, while at the intermediate and highest tableting pressure, all compositions gave similar tablet porosity. As expected, a decreased porosity of the tablets increased their tensile strength (Fig. 6a-b) and for all compositions, the compactibility was slightly higher for the mini-tablets produced at 50 MPa compared to those produced at 100 MPa.

Also as expected, an increased tableting pressure increased generally the tablet tensile strength (Fig. 6c-d). For all compositions, the tabletability was slightly higher for the mini-tablets produced at 50 MPa compared to those produced at 100 MPa. For both mini-tablet porosities, an increased *f*_MCC_ up to 75% *f*_MCC_ increased the tablet tensile strength at each investigated compaction pressure but the tablets made from only MCC mini-tablets (100% *f*_MCC_) had a lower tensile strength compared to tablets made of a 75% *f*_MCC_ mixture. Thus, the trend towards increased tablet tensile strength with increased proportion of MCC was broken at 100% *f*_MCC_, a finding that is not consistent with an earlier study on the tabletability of dry granulated particles (Tofiq et al., 2022a).

## 4 Discussion

### 4.1 Plasticity parameters

#### 4.1.1 Single mini-tablets

Since dry granulated particles are typically irregular in shape it is difficult to determine their mechanical properties through single particle analysis. In order to circumvent this problem, mini-tablets and flat faced tablets were used in this study as surrogates for the granules, i.e. two types of tablets of well-defined geometry. If these reference tablets (surrogate granules) have similar porosity as typical irregularly shaped granules formed by compaction as in a dry granulation technology, one can assume that the internal microstructure is similar and the surrogate tablets are in this regard representative. However, they are not resembling the external structure of irregular granules in two regards: First, the shape and surface rugosity is different and secondly, since the external surfaces of the mini-tablets were in contact with the tooling during preparation one can expect a locally higher relative density at the surface. In order to get an indication of the relative importance of internal and external structure for the compression behavior and tabletability, a comparison with earlier published compression and compaction data (Tofiq et al., 2022a; Tofiq et al., 2022b) using dry granulated particles of the same composition and porosity range was done. In addition, the mini-tablets used here have a considerably larger size and thus mass than what is typical for dry granulated particles. However, the yield pressures (Table 1) calculated from the linear part of the Heckel plots are similar as the yield pressures for some dry granulated particles of the same compositions studied earlier (Tofiq et al., 2022b) but with much lower diameters. Thus, the compression behavior of the mini-tablets was not considerably different to smaller dry granulated particles.

As expected, the *F*_g axial_ increased with a higher proportion of MCC in the composition. Although mini-tablets of defined and consistent size and shape were used for these measurements the variability in fracture strength *F*_g axial_ (Table 1) was relatively high and showed a dependence on the granule composition, i.e. the relative standard deviation increased with an increased proportion of LAC. Since a higher proportion of LAC is expected to result in more brittle granules, the increased variability can be attributed to an increased brittleness. In addition, the granule plastic deformation parameter also showed a high variability, which also increased with a higher proportion of LAC.

The *τ*_0_ parameter showed a positive correlation to the axial fracture force (*F*_g axial_) (Table 1) supporting that this compression parameter represents an indication of the fracture strength of granulated particles.

#### 4.1.2 Indentation test tablets

The use of indentation experiments for pharmaceutical applications has been notably scarce in the literature (Wilms & Kleinebudde, 2020). However, within the context of dry granulation by roller compaction, indentation experiments have been conducted to characterize ribbon indentation hardness (Al Asady et al., 2016; Freitag et al., 2004; Gago & Kleinebudde, 2017), to establish correlations between material indentation hardness and ribbon strength (Al Asady et al., 2015), and to investigate the spatial heterogeneities of ribbons (Miguélez-Morán et al., 2009). Indentation tests can be classified as macro-, micro-, and nano-scale techniques depending on the size of the indenter. Mostly, micro- and nanoindentation techniques have been used in a pharmaceutical context. Since granule plastic deformation has been shown to be associated with a macroscopic granule shape change during tableting (Tofiq et al., 2025) a macroscale indentation test was used in this study in order to derive an indication of the global granule indentation hardness. Moreover, macroscopic indentation hardness has been stated to be influenced by both the hardness of the starting materials and the bonding between the particles (Al Asady et al., 2015) and both these variables may affect also the plastic deformation of granules during compression. Thus, the measurement conditions used here to obtain indentation hardness data (*HB*) are considered to give indications of the plastic stiffness of the global granule structure rather than just the plastic stiffness of the solid material from which the granules were formed (the primary particles). Therefore, an indenter of a relatively large diameter was used, i.e., a ball-shaped indenter with a diameter of 5 mm. The indenter was subjected to a load of two-thirds of the strength of the compacts, resulting in dent diameters ranging between 0.6 and 2 mm, depending on the composition of the compacts, i.e., a compact of higher strength gave an increased dent diameter (Fig. 2).

The *HB* (Table 1) showed a positive correlation with the plastic deformation module (Fig. 1b). Thus, it is unexpectedly concluded that a higher proportion of MCC resulted in less deformable granules, as assessed by both these single specimen tests. The same finding has also been reported (Sun et al., 2018) for compacts of MCC and anhydrous dibasic calcium phosphate below a critical pressure of 150 MPa. Since both the indentation hardness and the plastic deformation module gave similar indications of granule plastic stiffness (Fig. 1b), and the variability of *HB* was relatively low, the latter is henceforth used as the indicator of single granule plasticity in the comparison to the powder compression parameters.

It has previously been reported (Gago & Kleinebudde, 2017) that a higher proportion of MCC mixed with mannitol, a substance considered to be brittle, decreased the micro-indentation hardness of ribbons prepared by roller compaction. The authors used a micro-indentation technique equipped with a ball indenter with a diameter of 0.4 mm. The different progressions in indentation hardness with a higher proportion of MCC compared to the findings in this work may be explained by the different indentation techniques used in the studies, i.e., micro-hardness vs. macro-hardness indentation techniques.

#### 4.1.3 Compression coefficient of minitablets

In order to calculate an indication of the plasticity of the mini-tablets during compression the equation derived by Adams and co-workers (Adams et al., 1994) is used. More specifically, the pressure or friction coefficient *α* in the equation is here used a measure of mini-tablet plastic stiffness. The coefficient, referred to by the authors as a pressure or friction coefficient, was introduced in the equation as an indication of how much the granule strength depends on the confining pressure. If one assumes that granule plastic deformation is the dominating response of the granules to the applied pressure in the macroscopic stiffening phase, an increased coefficient corresponds to an increased shear strength of the granules, i.e. an increased internal friction. The variability in coefficient between different granules can accordingly be explained by differences in the rate of increase in shear strength of the granule with increased applied pressure. A high rate of increase in resistance will decrease the total degree of plastic deformation of the granules during compression and is affected by the properties of the granule forming materials and the available space for shearing (porosity). We here use inverted values of the coefficient meaning that an increased inverted value corresponds to a reduced internal friction, here described as an increased plasticity or decreased plastic stiffness.

An increased concentration of MCC in the mini-tablets increased the *α*^−1^ coefficient, i.e., indicating a decreased plastic stiffness, which is consistent with an earlier finding for irregular dry granulated particles produced by slugging (Tofiq et al., 2022a). Mini-tablets with higher porosity gave mostly lower *α*^−1^ coefficients (Table 1), i.e., increased porosity resulted in more deformable mini-tablets. For granules with high LAC content, similar *α*^−1^ coefficients were obtained for both low and high porosity mini-tablets, indicating similar levels of plasticity. Although referred to as high and low porosity mini-tablets, the porosity difference was small for these mini-tablets (Table 1).

#### 4.1.4 Relationship between indentation hardness and inverted Adams coefficient

**Fig. 7:**
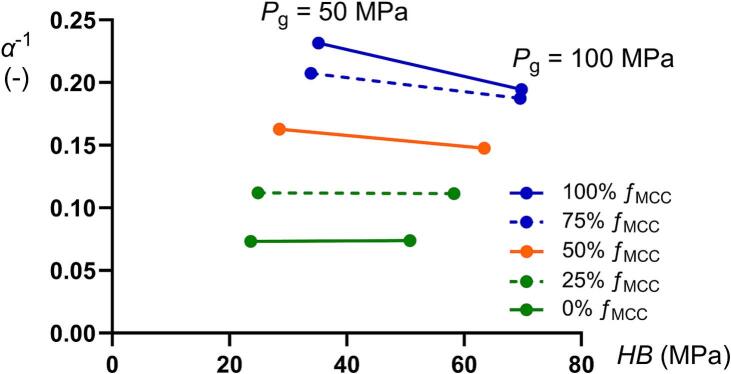
The inverted Adams coefficient *α*^−1^ as a function of the indentation hardness (*HB*) for all compositions compacted at 50 and 100 MPa.

There was no general correlation obtained between the *HB* and *α*^−1^ (Fig. 7). Given that the *α*^−1^ coefficient is a descriptor of the degree of plastic deformation of the mini-tablets which is expressed during powder compression, this powder compression property did not correlate to the indentation hardness or the deformability of the global mini-tablets structure when loaded as a single specimen. During powder compression, the plastic deformation of granules is associated with a permanent change in macroscopic granule shape (Tofiq et al., 2025), which is also described previously for pellets formed by wet granulation (Johansson & Alderborn, 1996). Such a shape change requires the rearrangement and flow of primary particles forming the granules, i.e., an internal shearing of the granules. Based on the progression of the stiffening region in the strain-pressure relationships (Tofiq et al., 2025), and thus the *α*^−1^ coefficient, we conclude that a higher proportion of MCC particles makes the granules more plastically deformable, although the converse applied for the plasticity as assessed by single mini-tablet loading and indentation of the indentation test tablets. It seems thus that the plastic deformation of the primary particles had different effects on the plastic deformation of the granules while loaded as single granules in unconfined state and while loaded as an assembly of granules in a confined state. Thus, during powder compression, when the granules are highly constrained within the die, it can be hypothesized that the global granule plastic deformation during compression is highly dependent on the plastic deformation of the primary particles, i.e., primary particle plastic deformation enables the shearing involved in granule plastic deformation during constrained condition in the die. For the single specimen tests, the bonding between the primary particles may resist plastic deformation giving a more complex relationship between primary particle plastic deformation and granule plastic deformation.

#### 4.1.5 Relationship between inverted Adams coefficient and Heckel yield pressure

The most common compression equation used in pharmaceutical science is probably the Heckel equation which was derived based on a kinetic model, i.e. the assumption that the compression can be described as a first order reaction with the pores as reactants (Heckel, 1961). The rate parameter of the equation is typically calculated from a linear part of the compression profile and the inverse parameter, typically referred to as the yield pressure, is stated to be a reliable indication of the single particle plasticity during powder compression (Vreeman & Sun, 2022). In contrast to powders of dense, non-porous particles, powders consisting of granulated particles (i.e. particles with some intragranular porosity) have a dualistic pore structure consisting of both inter- and intra-granular pores. In order to assess the plastic deformation of the granules, the relevant reactant pores are the inter-granular pores which in practice cannot be determined. Thus, the Heckel approach cannot be used to derive a compression parameter indicating the plasticity of granules (Johansson & Alderborn, 1996; Persson et al., 2016). In this study, we hence use the Heckel data treatment as a means to determine the yield pressure as a measure of the plastic stiffness of the primary particles.

**Fig. 8:**
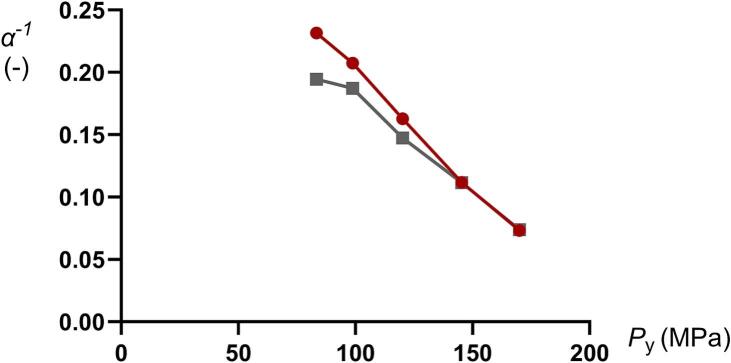
The Adams *α*^−1^ coefficient as a function of the Heckel yield pressure *P*_y_ (Tofiq et al., 2022b) for all compositions compacted at 50 (red dots) and 100 MPa (gray squares). (For interpretation of the references to colour in this figure legend, the reader is referred to the web version of this article.)

Given that the Heckel yield pressure (*P*_y_) represents an indication of the plasticity of the fine particles forming the granules, and that the inverted Adams *α*^−1^ coefficient is an indication of the granule plastic stiffness, one might expect a relationship between *P*_y_ and *α*^−1^ as discussed above (Fig. 8). For mini-tablets of both high and low porosity, the *α*^−1^ correlated nearly linearly to *P*_y_. For the mini-tablets consisting of 0% *f*_MCC_ and 25% *f*_MCC_, their plasticity was independent of their porosity (Table 1) while with increased *f*_MCC_ the relationships diverged, i.e., the effect of porosity on the plasticity of the mini-tablets became more pronounced when the mini-tablets became softer due to an increased proportion of *f*_MCC_. The relationships between the *α*^−1^ and *P*_y_ thus support the hypothesis that a causal relationship exists between the plasticity of the starting material and the plasticity of granular materials during powder compression prepared by dry granulation.

The same dependency of *P*_y_ for the plasticity was not obtained when indications of plasticity were derived from the single specimen tests. Since the latter mechanical parameters did not show the same dependency on primary particle plastic deformation, they are proposed to also depend markedly on the bonding between the primary particles of the granules.

### 4.2 Relationships between plasticity parameters and tablet tensile strength

The tensile strength of tablets has been proposed to depend on the strength and the area of the inter-particle bonds (Nyström et al., 1993). Given that the strength of a tablet formed from granulated particles is to a significant degree controlled by the strength and the area of the inter-granular bonds, the extent of plastic deformation of the granules during compression are consequently critical for their tabletability (Tofiq et al., 2022a). It is thus of interest to study the relationship between the tensile strength formed of the mini-tablets and their plasticity.

The tensile strength of the tablets increased with *f*_MCC_ up to a fraction of 75% *f*_MCC_ (Fig. 6c-d). Hence, the addition of the plastic component MCC increased the tablet tensile strength, in accordance with earlier reports on dry granulated particles (Perez-Gandarillas et al., 2015; Sun & Kleinebudde, 2016). In addition, the tabletability of the mini-tablets formed at the lower production pressure (more porous mini-tablets) was generally higher compared to the mini-tablets formed at the higher production pressure (Al Asady et al., 2018; Freitag et al., 2004; Wilms & Kleinebudde, 2020).

**Fig. 9:**
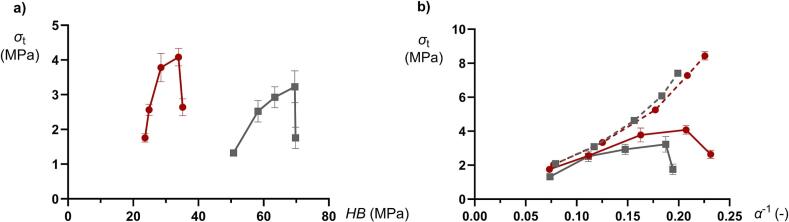
The tablet tensile strength at compaction pressure 300 MPa as a function of a) indentation hardness (*HB*) and b) inverted Adams coefficient *α*^−1^ for compositions prepared at 50 (red dots) and 100 MPa (gray squares). In b) data for dry granulated particles of a size fraction 500–710 μm (Tofiq et al., 2022b) are added for comparison (dashed lines). The bars denote the standard deviations. (For interpretation of the references to colour in this figure legend, the reader is referred to the web version of this article.)

The indentation hardness (Fig. 9a) and the inverted Adams *α*^−1^ coefficient (Fig. 9b) gave different relationships to the tablet tensile strength. An increased *HB* corresponded for each tablet porosity to an increased tablet tensile strength expect at the highest *HB*. Thus, contrary to what is typically expected a decreased plasticity of the indentation test tablets corresponded to an increased tablet tensile strength up to a fraction of 75% *f*_MCC_. Moreover, since the porosity of the indentation test tablets gave completely separated *HB* - *σ*_t_ relationships there was no tendency to a general relationship between *HB* and *σ*_t_. For the *α*^−1^ coefficient a markedly different pattern was obtained. First, the tablet strength increased with an increased coefficient, i.e., an increased compression plasticity, up to up to a fraction of 75% *f*_MCC_. Thus, a conflicting effect of plasticity was obtained between the two methods used to assess plastic deformation. Secondly, except for mini-tablets of 100% *f*_MCC_, the two *α*^−1^ - *σ*_t_ relationships for the respective mini-tablet porosity nearly coincided into a single relationship. However, a slightly higher gradient of this relationship was obtained for the more porous mini-tablets. Since there were no general relationship between granule indentation hardness, as assessed by macro-indentation, and the tablet tensile strength, the use of macro-hardness indentation data, which is here considered to represent the hardness of the global granule structure, may be misleading in predicting the tabletability of dry granulated particles.

Based on the relationship between tablet tensile strength and the inverted Adams coefficient *α*^−1^ (Fig. 9b), we propose that plastic deformation of the mini-tablets is the main compression mechanism controlling their tabletability. The plastic deformation of the mini-tablets will control the development of intimate contact between them and thus the formation of strong inter-granular bonds due to a large bonding area. The trend with an increased tablet tensile strength with increased plastic deformation was however broken for mini-tablets of 100% *f*_MCC_ (Fig. 9b). An explanation for this decrease in tabletability is that due to a high indentation hardness of the mini-tablets in combination with their regular shape the local plastic deformation at the junctions between the mini-tablets is reduced and the development of large inter-granular areas decreased. An alternative explanation is that some degree of granule fragmentation or attrition may be required at the inter-granular junctions in order to fully utilize the potential of granule plastic deformation for inter-granular bonding. The formation of such intercalated fragments or primary particles may increase the area of inter-granular bonds. In the absence of these, i.e., for granules with very high fracture strength, the tabletability may decrease. Thus, to fully understand the tabletability of dry granulated particles, both granule fragmentation and granule plastic deformation should be considered, but the crucial mechanism is here proposed to be plastic deformation.

An intriguing question concerns the role of fragmentation and plastic deformation for the loss of tabletability (LoT) and for the absolute tabletability, respectively. It is earlier proposed (Tofiq et al., 2025) that the degree of fragmentation is the major process dictating the loss of tabletability, while plastic deformation has a secondary importance. However, it is suggested here that the absolute tabletability is controlled predominantly by the degree of granule plastic deformation, while fragmentation has a secondary importance. Both these mechanisms will hence be of relevance to indicate tableting performance in a broad sense, as also proposed earlier (Tofiq et al., 2022a). The concept of LoT refers to a comparison of the tensile strength of tablets formed from powders of the same composition but with particles of a considerably different size and density. These two types of tablets will typically differ substantially in microstructure, i.e., the dimensions of particles, voids, and pores of the tablet. However, a high degree of granule fragmentation during compression will result in a greater similarity in microstructure and consequently a low LoT. The absolute tabletability, on the other hand, involves the comparison of tabletability of granules of similar size and shape but of different compositions, porosity, and strength. In this relative comparison, the degree of plastic deformation in granules will cause differences in critical microstructural features of the tablet and, consequently, the tablet tensile strength. Previous studies have shown (Skelbæk-Pedersen et al., 2021; Tofiq et al., 2025) that a considerable fraction of granules will survive the compaction in terms of having a size resembling the original uncompressed granules. These granules may, however, be deformed in different ways in terms of mode and degree. The plastic deformation of these relatively large granules may be critical in controlling the area of contact developed between a granule and its surrounding particles, which could be other nearly intact granules or granule fragments of different sizes, and thus tablet tensile strength.

### 4.3 Comparison of spherical granules (mini-tablets) and irregular shaped granules

In Fig. 9b, the earlier presented relationship between tensile strength and *α*^−1^ for dry granulated particles prepared by slugging-milling (Tofiq et al., 2022a) is plotted in the same graph as the relationship obtained in this study for the mini-tablets. For the less deformable granular solids, which also show the highest degree of fragmentation (Tofiq et al., 2025), the relationships for the two types of granular material coincide. However, for the more deformable granular material, from 50% *f*_MCC_ and up-wards, the respective relationships between tensile strength and *α*^−1^ deviate from each other and the dry granulated particles showed a higher tabletability.

The relationship between the *α*^−1^ coefficients for mini-tablets and dry granulated particles respectively was linear as discussed above (Fig. 5), indicating that the internal micro-structure of the granular materials dictates their plastic deformation independent of a difference in external structure. The plastic deformation phase is preceded with a fragmentation-rearrangement phase which is affected by both the internal and external micro-structure (Fig. 4). In this first phase, the compressibility was dependent on the degree of fragmentation of the granular materials. For granules showing low degree of fragmentation, a more irregular shape and surface structure facilitated the compression compared to the mini-tablets while the converse applied for granules showing a high degree of fragmentation. In this case, it seems that the type of fragmentation shown by the mini-tablets facilitated the subsequent rearrangement and compressibility. Also the tabletability was affected by both internal and external micro-structure of the granular materials dependent on the fragmentation and hardness of the granular material.

## 5 Conclusions

In this study, we first aimed to investigate if there is a correlation between mechanical parameters derived through single granule testing and compression parameters derived through analytical powder compression and secondly, if any of these parameters are linked to the tabletability of the granules. It is found that:

1. The effective plastic deformation expressed during powder compression, as indicated by the inverted Adams coefficient *α*^−1^, did not correlate with the plasticity of the single surrogate tablets as measured by uniaxial loading and indentation. It was further found that the plasticity of the primary particles forming the mini-tablets was critical for the degree of granule plastic deformation which is expressed during confined compression which was however not the case for plasticity parameters derived from single specimen mechanical tests.
2. A good correlation was obtained between the inverted Adams coefficient *α*^−1^ and the tensile strength formed of the mini-tablets, supporting the critical role of granule plastic deformation expressed during powder compression for their tabletability. There was consequently no correlation between tablet tensile strength and the indentation hardness of the indentation test tablets. Moreover, mini-tablets of high strength deviated from the general progression of the relationship between tablet tensile strength and *α*^−1^, exhibiting an unexpectedly low tabletability. There is, thus, a need for local adjustment of contacts between granules, for example, through intercalated granule fragments.

Since the compositions of the mini-tablets was the same as used earlier for dry granulated particles it was possible to compare the tabletability and the *α*^−1^ coefficient – tablet tensile strength relationships between mini-tablets and dry granulated particles. It was found that the internal micro-structure of the granular materials dictated their plastic deformation independent of a difference in external structure. However, the internal and the external micro-structure jointly affected the compressibility and the tabletability of the granular materials.

In summary, we conclude that granule plastic deformation expressed during powder compression is a key property influencing the tabletability of granulated particles. Formulation strategies on how to modulate the plastic stiffness of the granules through changes in their composition need to be further investigated. Moreover, proper assessment of the effective plastic deformation of granules is an important tool in formulating powders intended for dry granulation. We propose that analytical powder compression is the preferred approach for such assessments.

## CRediT authorship contribution statement

**Maryam Tofiq**: Conceptualization, Methodology, Validation, Investigation, Writing – Original draft, Review and editing.

**Göran Alderborn**: Conceptualization, Resources, Methodology, Validation, Writing – Review and editing.

**Josefina Nordström**: Conceptualization, Methodology, Writing – Review and editing.

**Ann-Sofie Persson**: Conceptualization, Methodology, Investigation, Validation, Writing – Original draft, Review and editing.

## Declaration of competing interest

The authors declare the following financial interests/personal relationships which may be considered as potential competing interests:(Maryam Tofiq reports financial support was provided by Sweden's Innovation Agency. If there are other authors, they declare that they have no known competing financial interests or personal relationships that could have appeared to influence the work reported in this paper.)

## Data Availability

Data will be made available on request.
